# Immune modulation by helminth parasites of ruminants: implications for vaccine development and host immune competence[Fn FN1]

**DOI:** 10.1051/parasite/2014051

**Published:** 2014-10-09

**Authors:** Tom N. McNeilly, Alasdair J. Nisbet

**Affiliations:** 1 Disease Control, Moredun Research Institute, Pentlands Science Park EH26 OPZ UK; 2 Vaccines and Diagnostics, Moredun Research Institute, Pentlands Science Park EH26 OPZ UK

**Keywords:** Helminths, Immune modulation, Ruminant, Vaccination, Coinfection

## Abstract

Parasitic helminths reside in immunologically-exposed extracellular locations within their hosts, yet they are capable of surviving for extended periods. To enable this survival, these parasites have developed complex and multifaceted mechanisms to subvert or suppress host immunity. This review summarises current knowledge of immune modulation by helminth parasites of ruminants and the parasite-derived molecules involved in driving this modulation. Such immunomodulatory molecules have considerable promise as vaccine targets, as neutralisation of their function is predicted to enhance anti-parasite immunity and, as such, current knowledge in this area is presented herein. Furthermore, we summarise current evidence that, as well as affecting parasite-specific immunity, immune modulation by these parasites may also affect the ability of ruminant hosts to control concurrent diseases or mount effective responses to vaccination.

## Abstract (fr)

Les helminthes parasites résident dans des localisations extracellulaires immunologiquement exposées au sein de leurs hôtes, mais sont pourtant capables d’y survivre pendant de longues périodes. Pour permettre cette survie, ces parasites ont développé des mécanismes complexes et multiformes de subversion ou de suppression de l’immunité de l’hôte. Cette synthèse fait un résumé des connaissances actuelles sur la modulation immunitaire par les helminthes de ruminants et les molécules dérivées des parasites qui sont impliquées dans cette modulation. De telles molécules immunomodulatrices sont très prometteuses en tant que cibles de la vaccination, puisqu’on prédit que la neutralisation de leur fonction améliorera l’immunité anti-parasite. Les connaissances actuelles dans ce domaine sont présentées ici. De plus, nous résumons les preuves actuelles que la modulation immunitaire par ces parasites peut, en plus d’affecter l’immunité spécifique au parasite, diminuer la capacité des ruminants hôtes à lutter contre les autres maladies ou à mettre en place des réponses efficaces à la vaccination.

## Introduction

Helminths are complex multicellular eukaryotic parasites which infect a large proportion of the global human and livestock population. They are comprised of two highly divergent taxa, the roundworms (nematodes) and the flatworms (trematodes and cestodes). Despite this divergence, the immune response generated against roundworms and flatworms is broadly similar, being characterised by T-helper 2 (Th2) immune responses involving the cytokines interleukin-4 (IL-4), IL-5 and IL-13, immunoglobulin (Ig) E, and specialised effector cells such as mast cells, eosinophils and basophils [48]. Given their large size, these parasites are unable to penetrate host cells and instead reside in extracellular sites such as the lymphatics, bloodstream, tissues or organs where they are exposed to the host immune response. In order to avoid expulsion from the host, helminth parasites have evolved highly elaborate and complex mechanisms to suppress or subvert the mammalian immune response [48, 54]. These modulatory mechanisms are not specifically targeted to the host’s anti-parasite response, as responses to bystander antigens are also affected during helminth infection [45]. Curative chemotherapy has been shown to restore normal immunological function *in vivo* [78] suggesting that modulation of the host immune response is an active process, requiring the parasites to release factors into their environment which interact with host immune cells and molecules. As this release could involve active export of molecules via secretory pathways, or passive diffusion of molecules from the parasite soma, products released from the parasite are currently termed “excretory-secretory (ES)” products, and significant efforts have been made to identify and characterise immunomodulatory activity within parasite ES products released by the parasites.

Whilst the majority of data relating to helminth immune modulation has been obtained from rodent and human studies, there is increasing evidence that certain ruminant helminths are similarly capable of modulating host immune responses. Such immune modulation potentially explains why immunity to ruminant helminth parasites is so slow to develop and is often poorly protective [3, 21, 44], but may also have a number of important consequences for livestock health and welfare. Firstly, immunomodulatory molecules may be useful targets for parasite vaccine or therapeutic development, as they represent molecules which are of importance for parasite survival within the host. Secondly, modulation of the immune response by helminths may impact on the susceptibility of livestock to other infectious diseases, and may also impact on responses to other livestock vaccines.

This review will summarise current knowledge of immune modulation by helminth parasites of ruminants, focussing on the nematodes and trematodes of economic importance to the livestock industry, defining what progress is being made on the use of helminth immunomodulators in anti-parasite vaccines and what impact these helminth infections have on immune competence of the ruminant host.

## Immune modulation by trematode parasites of ruminants

Of the trematodes affecting global ruminant populations, the liver fluke, *Fasciola hepatica*, is one of the most important. Following ingestion of encysted dormant larvae, infective juveniles traverse the duodenal wall and enter the peritoneal cavity where they migrate to, and penetrate, the liver capsule. Thereafter, the juvenile larvae migrate through the liver parenchyma before moving to the bile ducts where they mature into adult stage parasites [3]. The invasive nature of the parasite lifecycle results in significant exposure to the host immune response, both humoral and cellular. Despite this, the parasites are able to survive for extended periods in the host (usually 1–2 years in cattle and up to 20 years in sheep [3]), and while *F. hepatica* induces potent and highly polarised Th2 immunity in the host, these responses do not appear to be protective: indeed the magnitude of the Th2 responses is positively correlated with parasite burden [13]. Protection against liver fluke infections can be achieved by vaccination and is dependent on induction of Th1-type immunity [32, 56, 57]. Thus, by driving Th2 immunity, the parasite can down-regulate protective Th1 immunity, enabling its survival within the host. In addition to skewing the immune response towards non-protective Th2, the parasite is also capable of generating regulatory immune responses: following penetration of the intestinal wall by the newly-excysted juvenile flukes, peritoneal macrophages display markers of regulatory/M2 macrophages (e.g. arginase-1 and PD-1) and secrete the regulatory cytokines IL-10 and transforming growth factor-beta (TGF-β) [18, 20], and regulatory rather than Th2 responses predominate during later stages of infection, with a reduction in parasite-driven IL-4 synthesis but increased IL-10 and TGF-β production [28].

*F. hepatica* ES products (FhES) have been shown to possess immunomodulatory properties both *in vitro* and *in vivo*. For example, FhES is capable of suppressing concanavalin A-induced proliferation of ovine lymphocytes [41, 69] and skews the phenotype of both ovine and bovine macrophages towards a regulatory/M2 phenotype [25, 27], whereas systemic administration of FhES to mice has been shown to prevent development of Th1 immunity following immunisation with killed *Bordetella pertussis* vaccines [65]. Given the similarities of the immunomodulatory effects of FhES and those seen during parasite infection *in vivo*, significant efforts have been made to identify specific immunomodulatory molecules within FhES, with some notable successes. As the immunomodulatory molecules secreted by *F. hepatica* have been extensively reviewed elsewhere recently [16, 71, 72], only a brief overview of these molecules is provided below.

### Peroxiredoxin (FhPrx)

The anti-oxidant enzyme peroxiredoxin (Prx) derived from *F. hepatica* ES products (FhPrx) may protect the parasite by inactivation of host reactive oxygen species (ROS) but has also been shown to induce the production of alternatively-activated macrophages (AAMΦs) in mouse models, thus potentially promoting the development of host Th2 responses when it is secreted by flukes resident in ruminants [18, 20]. Further evidence for this latter mechanism was provided by the inhibition of Th2-like responses in *F. hepatica*-infected mice which had received ovine anti-FhPrx antibodies by passive transfer (reviewed in [16]).

### Cathepsin L cysteine proteases (FhCL)

The synthesis and release of cysteine proteinases is an integral part of liver fluke lifestyle and immunomodulation – emergence of the juvenile flukes from the ingested cyst is dependent on the synthesis of cysteine proteinases and the subsequent release of such molecules may also cleave host immunoglobulins, preventing antibody-mediated killing mechanisms from operating at this early stage in infection [10, 74]. A large proportion (up to 80%) of the ES material produced by liver flukes (FhES) consists of cathepsin L cysteine proteinases [16], and the suppressive effects of FhES on ovine lymphocyte proliferation and *Bordetella pertussis* vaccine-induced interferon-gamma (IFN-γ) responses are largely associated with cysteine protease activity [65, 69]. *Fasciola hepatica* cathepsin L1 (FhCL1), which is a key vaccine candidate molecule (see Section on Helminth immunomodulators as vaccine candidates, below) secreted by all stages of the parasite, has immunomodulatory activity through inhibiting the release of pro-inflammatory factors from macrophages by cleavage of toll-like receptor (TLR) 3 within the macrophage endosome [19]. In addition, *Fasciola hepatica* cathepsin L5 (FhCL5) can interfere with T cell function via cleavage of cell surface CD4 [69].

### Cathelicidin-like helminth defence molecules

Helminth defence molecules (HDMs) are non-cytotoxic, non-bacteriocidal functional mimics of mammalian cathelicidin-like host defence peptides [72]. Following proteolytic processing by FhCL1, an *F. hepatica* HDM (FhHDM-1) is able to bind bacterial lipopolysaccharide (LPS), preventing its interaction with TLR4 on macrophages and thus inhibiting innate immune responses [73]. In addition, following internalisation of FhHDM-1 into macrophage endosomes, the molecule can inhibit antigen degradation and thus impair antigen presentation via MHC class II [70].

## Immune modulation by parasitic nematodes of ruminants

Gastro-intestinal nematodes (GIN) are the most important nematodes affecting ruminant species in terms of lost productivity. A common feature of infections with these nematodes is that immunity takes a high proportion of the productive lifespan of the animal to develop. For instance immunity to *Ostertagia ostertagi*, the most important nematode parasite affecting cattle, is usually only acquired in the second grazing season [21], whereas immunity to *Teladorsagia circumcincta*, an important GIN of small ruminants in temperate regions, only develops after constant exposure to the parasite over a number of weeks [81]. Furthermore, immunity to these parasites is often incomplete, requiring constant exposure of the parasite for its maintenance [83], and rarely being completely protective against re-infection [44]. The slow development and weak nature of the immune response suggests that these parasites are actively down-regulating the host immune response during infection.

Ruminant GIN possess a direct and relatively simple lifecycle. Following ingestion of infective stage larvae, the parasite undergoes additional developmental changes within, or closely associated with, the gastro-intestinal mucosa before development into adult-stage parasites in the gastro-intestinal lumen. Thus particular developmental stages of the parasite, for example mucosal fourth-stage larvae (L4) of *O. ostertagi* and *T. circumcincta*, are more intimately associated with the host, and evidence suggests that these parasitic stages may be particularly effective at modulating host immunity. For example, soluble somatic extracts and ES products from *O. ostertagi* L4 but not extracts from L3 or adult stage parasites are able to suppress T cell proliferation *in vitro*, with such effects involving up-regulation of the regulatory cytokines IL-10 and TGF-β [35]. Similarly, ES products from L4 of *T. circumcincta* are capable of suppressing ovine T cell activation *in vitro*, again involving up-regulation of IL-10 [52]. Interestingly, L4 ES products from two other small ruminant gastro-intestinal nematodes (GIN), *Nematodirus battus* and *Trichostrongylus vitrinus*, are unable to suppress T cell activation (McNeilly, unpublished observation), indicating that different ruminant GIN may possess different levels of immunomodulatory capacity. This would be consistent with studies in sheep where the development of protective immunity through repeated parasite challenge is more easily achievable with the GIN *Haemonchus contortus* and *Trichostrongylus colubriformis* than with *T. circumcincta* [39]. It may also indicate that different ruminant GIN have evolved different mechanisms to evade host immunity. Taken together, current evidence would suggest that certain ruminant nematode species are effective at modulating mammalian host immunity. While the full range of immunomodulatory molecules produced by these parasites remains to be determined, molecular and proteomic approaches have so far identified a number of ruminant nematode molecules with putative or demonstrable immunomodulatory function. These are listed below.

### Macrophage migration inhibitory factor (MIF)

Macrophages have diverse roles in immunity to pathogens including antigen presentation, phagocytosis and killing following “classical” activation [1] and additional roles in immune responses to helminth infection following “alternative” activation [36]. Although macrophages are not thought to possess nematode antigen-specific receptors, they do colocalise with lymphocytes at sites of nematode infection [50] and can be involved in the immobilisation of tissue-migratory nematodes through nematode-specific antibody-dependent alternative activation in immune hosts [22]. Interference with macrophage motility and function could therefore be a mechanism by which parasitic nematodes evade the host’s immune system.

Macrophage migration inhibitory factor (MIF) is a cytokine which, in mammals, is synthesised by a variety of cells including T lymphocytes, endothelial cells, fibroblasts and monocytes/macrophages [9]. MIF inhibits the random migration of monocytes/macrophages, influences cytokine production, upregulates TLR4 expression in immune cells and acts as an important pro-inflammatory cytokine in innate immunity [5, 9, 75]. A number of parasitic nematodes also produce MIF orthologues (reviewed in [95]) which have the potential to influence host macrophage cell function and host immune response development. This interference could be through inhibiting the movement of sensitised macrophage antigen-presenting cells (APCs) from the local tissue which the worm occupies or through the induction or antagonism of specific host inflammatory/anti-inflammatory pathways [95]. An example of the latter process has been demonstrated in mouse models of filarial nematode infection where *Brugia malayi* MIFs (Bm-MIFs) induced AAMΦs *in vivo* [23, 68] and the continuous release of Bm*-*MIFs by adult worms is thought to activate an anti-inflammatory pathway, favouring worm survival [50].

In parasitic nematodes of ruminants, the presence of an active MIF orthologue (Tci-MIF-1) has been demonstrated in extracts of larval *T. circumcincta* and the molecule has been localised to the gut of *T. circumcincta* parasitic stages suggesting that Tci-MIF-1 may be excreted to influence monocyte movement and activation in the host abomasum [60]. Homology searching of the transcriptomic datasets of other Clade V nematodes with the coding sequence (CDS) of Tci-MIF-1 in Nembase4 (http://www.nematodes.org/nembase4) reveals transcripts from *O. ostertagi* with up to 96% identity to the *Tci-mif-1* CDS across 310 nucleotide bases, and BLASTx searching of the NCBInr database (http://blast.ncbi.nlm.nih.gov/Blast.cgi) with the Tci-MIF-1 sequence also revealed a homologous MIF from *H. contortus* with 91% amino acid identity (96% similarity) across all 115 residues. These levels of identity demonstrate conservation of expression, and potentially function, across these parasitic nematodes of ruminants opening up the opportunity for cross-species protection with a multivalent vaccine if MIF is demonstrated to be a viable vaccine candidate (see Section on Helminth immunomodulators as vaccine candidates, below) for parasitic nematodes of ruminants.

### Apyrases

When mammalian tissues are injured, the processes involved in cell damage and cell death result in the presence of elevated levels of extracellular nucleotides, including adenosine 5′-triphosophate (eATP) which, as a “Danger-Associated Molecular Pattern” (DAMP) molecule, directly affects host immune responses, through, for example, the induction of profound pro-inflammatory processes such as the chemotaxis and degranulation of mast cells, neutrophils and eosinophils [7, 17, 90]. These types of responses are termed “purinergic” and result from the interaction of purines (e.g. eATP) with membrane purinoreceptors which have differential specificities for ATP and its metabolites adenosine 5′-diphosphate (ADP), adenosine 5′-monophosphate (AMP) and adenosine (90).

Apyrases (nucleoside triphosphate dephosphorylases, EC 3.6.1.5) are enzymes which hydrolyse the potent pro-inflammatory purine ATP to form ADP. Apyrases can then also hydrolyse ADP to AMP which may then be further hydrolysed by a 5′-nucleotidase to adenosine which exhibits anti-inflammatory capacity by inhibiting platelet aggregation, preventing amplification of the inflammatory response [37]. Some bloodfeeding insects exploit this mechanism by secreting a Ca^2+^-dependent apyrase in their saliva which hydrolyses purinergic nucleotides thus preventing clotting and permitting continued feeding [93]. Enzymes belonging to this group of Ca^2+^-dependent apyrases have also recently been described in both *O. ostertagi* (Oos-APY-1) and *T. circumcincta* (Tci-APY-1) [64, 100] and the enzymes from these two nematodes share 92% identity (96% similarity) across all 339 residues. BLASTx searching of the NCBInr database (http://blast.ncbi.nlm.nih.gov/Blast.cgi) with the Tci-APY-1 sequence also revealed three homologous apyrases with 65–71% amino acid identity (79–82% similarity) from *H. contortus*. In both *T. circumcincta* and *O ostertagi*, the production of these Ca^2+^-dependent apyrases was associated predominantly with fourth stage larvae (L4) (64,100) and Tci-APY-1 is present in the ES fluid of this stage of *T. circumcincta* [82]. Oos-APY-1 was originally identified by immunoscreening an *O. ostertagi* L4 cDNA library with sera from *O. ostertagi*-infected calves and, through Western blotting, it was apparent that the calves mounted a circulating antibody response to Oos-APY-1 within 2 weeks of infection [100]. In addition, immunolocalisation studies demonstrated localisation of Oos-APY-1 within the glandular bulb of the oesophagus at the oesophageal/intestinal junction [100]. Taken together, these results are highly suggestive of the secretory nature of Oos-APY-1 and Tci-APY-1 during the L4 stage of the parasites. It was therefore proposed that, by secreting apyrase and reducing levels of eATP locally, these nematodes could control inflammation within the gastric gland microenvironment, prolonging their longevity by immune evasion during a period of rapid larval growth while in intimate contact with the host tissue [64, 100]. This mechanism of immune evasion is consistent with studies of the intravascular stages of the blood fluke *Schistosoma mansoni* which express a panel of ATP-catabolising enzymes, including apyrase, on their tegumental surface, which are proposed to be involved with the conversion of host-derived eATP to adenosine [6]. *Trichinella spiralis* ES products also contain a cascade of nucleoside-metabolising enzymes which are thought to inhibit purinergic signalling and thus prolong nematode persistence within the host digestive tract [37, 38].

### Transforming growth factor-beta (TGF-β)-like molecules

TGF-β family members are evolutionarily-conserved cytokines involved in the regulation of an array of physiological systems and processes. In mammalian immune systems, TGF-β1 is the principal active isoform exerting regulatory effects on a variety of immune cells [47] with specific roles in T cell differentiation, development, homeostasis and tolerance (recently reviewed in [66]). In parasitic nematodes, homologues of TGF-β (termed TGHs) have principally been investigated for their potential roles in hypobiosis and diapause, largely because of their homology to the *Caenorhabditis elegans daf-7* molecule which is involved in dauer larvae formation [61]. However, one study, published in 2000, demonstrated the potential for a secreted TGF-β homologue from *Brugia malayi* adults (Bm-TGH-2), to bind mammalian TGF-β receptors leading to signal transduction in an *in vitro* system [34]. This raised the possibility that parasitic nematodes may have adapted TGF-β family genes to suppress the host immune system [49]. Subsequently, genes encoding TGF-β homologues have been identified from *H. contortus* and from *T. circumcincta* [53] with the expression of the latter being associated with the adult stage of the parasite. An intriguing possibility is, therefore, that *T. circumcincta* resident in the abomasum produce Tci-TGH-2 which ligates host TGF-β receptors to down-regulate protective immunity and/or pathology. One possible route to immune modulation by nematode TGH molecules is through the induction of host regulatory T cells (CD4^+^ Foxp3^+^ Tregs) which are induced from thymus-derived T cell precursors under the influence of TGF-β [43]. Infection of mice with parasitic nematodes induces host Treg cells [24, 31] and ovine Treg cells have also recently been demonstrated in the abomasal mucosa of *T. circumcincta*-infected sheep [51, 52], giving some indirect support for such a mechanism.

### Complement system derailing

The mammalian complement system is part of the innate immune system which assists in the anti-pathogen activities of antibodies and phagocytic cells. The system consists of three distinct pathways: the classical pathway, the alternative pathway and the lectin pathway. All three pathways rely on C3-convertase cleaving and activating component C3, causing a cascade of further cleavage and activation events [33]. During feeding, parasitic nematodes ingest host complement as well as host antibodies and these therefore have the potential to interact within the parasite causing tissue disruption. Studies focused on *H. contortus* over the last decade have identified some mechanisms by which this blood-feeding parasite modulates the host complement system to avoid these disruptive effects [76, 87, 88]. Calreticulin, a Ca^2+^ binding protein, is present in the ES material of adult *H. contortus* and inhibits the classical pathway by binding host C-reactive proteins and C1q – the component of the C1 complex which binds IgM or IgG to activate the classical pathway [87, 88]. Recently, a component of *H. contortus* adult ES was purified by affinity chromatography using immobilised goat C3 and was termed H.c-C3BP (*Haemonchus contortus* C3 binding protein) [76]. Proteomic analyses of this ES component suggested that it was a secreted glyceraldehyde-3-phosphate dehydrogenase (GAPDH), a ubiquitously-expressed gene in parasitic nematodes (e.g. [86]). Recombinant versions of this H.c-C3BP/GAPDH were bound by immunoglobulins in the sera of infected animals and displayed complement function inhibitory activity [76].

### Galectins

Galectins are proteins which bind sugars with a specific affinity for β-galactoside. Within the mammalian immune system, galectins can bind to the surface of parasitic helminths, as well as other pathogens, initiating host immune responses [94]. During experimental infections of immune sheep with *T. circumcincta*, for example, a host galectin-14 molecule is upregulated in the abomasal mucosa, potentially as part of a Th2 polarised immune response [30]. Parasitic nematodes of ruminants also produce galectin molecules in their ES material (e.g. see [14]) and immunomodulatory activity has been associated with these molecules. For example, lactose-affinity chromatography has been used to purify a mixture of galectins derived from larval *H. contortus* which, when mixed with ovine bone marrow eosinophils *in vitro*, exhibited eosinophil chemokinetic activity [91]. Recombinant versions of two isoforms of a galectin from *H. contortus* (termed *Hco*-gal-m and *Hco*-gal-f because they were derived from male and female worms, respectively) were able to induce apoptosis in goat peripheral blood mononuclear cells (PBMC) following binding to the cell surface [89]. This interaction was further elucidated through a recent proteomic and transcriptomic analysis which highlighted the ability of these recombinant galectins to activate components of both the TLR and CASPASE pathways along with down-regulation of pro-inflammatory cytokines in mitogen-stimulated PBMC [96].

## Other immunomodulatory molecules – routes for discovery

It is beyond the scope of this review to address all of the potential immunomodulators produced by parasitic helminths, but comprehensive reviews of this area have recently been published [42, 50]. These reviews, along with an increasing volume of original literature, highlight the numbers of immunomodulatory molecules being identified in filarial nematodes, schistosomes and from mouse models of helminth infection (e.g. [40]). In the case of filarial nematodes and schistosomes, these immunomodulators clearly have vital roles in the prolonged (years) survival of the parasites within the vascular and lymphatic systems of the hosts, and are therefore rich sources of information for those studying ruminant: helminth interactions to mine for homologous immunomodulatory molecules (e.g. proteinase inhibitors [4, 58]; recently reviewed by [55]). Analysis of immunomodulatory molecules in mouse models of helminth infection have shown that, in the case of the nematode *Heligmosomoides polygyrus*, adult worms produce powerful immunomodulatory molecules in their ES material, but immunisation of mice with this ES material leads to sterile immunity [40]. The identification of the immunomodulatory molecules in these cases, along with the increasing availability of the transcriptomes and genomes of helminth parasites of ruminants (e.g. the recently published *Haemonchus contortus* genome [46, 80] will assist greatly in assessing immunomodulators for use as vaccine candidates against ruminant parasitic helminths.

## Helminth immunomodulators as vaccine candidates

One of the main practical applications for exploiting helminth immunomodulators is in the development of vaccines to control parasitic infections. If immunomodulatory molecules can be delivered to the host in a manner which provokes a significant circulating antibody response (e.g. by injection with a suitable adjuvant) then, in theory, neutralising antibodies will nullify the immunomodulatory molecules released by the parasite during infection, leading to its exposure to the developing protective immune system and, ultimately, the parasite’s demise. One potential flaw in this process is that, by definition, it may be difficult to raise an immune response to an active immunosuppressive molecule and the specific activity of the antigen may need to be inactivated prior to use as a vaccine (e.g. see [4]). Some examples of the use of immunomodulators as vaccine antigens against monospecific helminth parasite infections of ruminants are described below.

### Teladorsagia circumcincta

Immunomodulatory molecules were identified through a combined proteomic/transcriptomic analysis of L4 *T. circumcincta* as part of a tripartite approach to identifying antigens for inclusion in a recombinant subunit vaccine cocktail [63]. These three molecules were an apyrase (Tci-APY-1; [64]), a macrophage migration inhibitory factor (Tci-MIF-1; [60]), and a TGF-β homologue (Tci-TGH-2; [53]). In addition, four ES proteins (a cathepsin F, a metalloproteinase, an activation-associated secretory protein, and an ES protein of unknown function) were selected by examining larval ES targets of local IgA responses in sheep rendered immune to re-infection [82]. An immunogenic homologue of a protective antigen of the canine hookworm, *Ancylostoma caninum* (*Ac*-SAA-1 [62, 101]) was also selected. Recombinant versions of each of these eight candidates were generated and combined into a sub-unit vaccine which was delivered, subcutaneously with the adjuvant Quil A, to sheep. The animals were subsequently subjected to a trickle challenge infection regime with *T. circumcincta* infective larvae over a period of 4 weeks and the trial was performed twice. In both trials, vaccinated sheep had significantly lower mean faecal egg counts (FEC) over the period of the experiment, with an overall mean FEC reduction of 72% (Trial 1) and 58% (Trial 2). During the peak egg shedding periods, vaccinated sheep shed 92% and 73% fewer eggs than control sheep in Trials 1 and 2, respectively. At post mortem, vaccinated sheep had 75% (Trial 1) and 57% (Trial 2) lower adult nematode burdens in the abomasum than those in the control group [63].

### Haemonchus contortus

Successful vaccination of sheep against haemonchosis has been achieved by immunising sheep with an *H. contortus* gut-derived membrane protein complex (H-gal-GP; [84]) which contains multiple antigens including proteinases and a galectin component [59]. However, affinity-purified galectin from the H-gal-GP complex did not confer protection against challenge infection in lambs when used individually as a vaccine in immunisation/challenge experiments [59]. Nevertheless, when goats were immunised with equal quantities of bacterially-expressed recombinant versions of the galectins *Hco*-gal-m and *Hco*-gal-f (100 μg of each per vaccination), subcutaneously with Freund’s adjuvants, and then challenged with a single infection of 5,000 infective L3, worm burdens were reduced at necropsy (399 ± 81 in the immunised goats compared with 742 ± 241 in the control, adjuvant only goats) with a corresponding reduction in cumulative faecal egg count [99].

### Fasciola hepatica

*F. hepatica* secretes several molecules with known immunomodulatory functions, for example cathepsin L1 (FhCL1), and peroxiredoxin (FhPrx) (see Section on Immune modulation by trematode parasites). Vaccination of sheep and cattle with native forms of cathepsin L1 resulted in reductions in fluke burdens in the range of 55–72% compared to unvaccinated controls (reviewed in [32]) and vaccination of cattle with a yeast-expressed recombinant version of FhCL1 an oil emulsion also led to a 48% reduction in fluke burden following field exposure [32]. Vaccination of goats with FhCL1 in Quil A gave less clear levels of protection with vaccinated goats having fluke burdens of 56 ± 26 compared with 92 ± 53 in adjuvant only controls following challenge [67]. The use of FhCL1 and FhPrx as a cocktail vaccine could potentially give additive or synergistic effects to the levels of protection, however goats co-immunised with yeast-expressed recombinant FhCL1 and bacterially-expressed recombinant FhPrx with Quil A adjuvant, and experimentally challenged with *F. hepatica* metacercariae, were not protected, despite developing high circulating antibody levels to both antigens [8].

## Impact of helminth infections on host immune competence

Given the potential impact of helminths on the ruminant immune response, there is increasing interest in defining what impact these parasites may have on general host immune competence as a result of bystander immune modulation (i.e. modulation of non-helminth-specific immunity). Studies have been broadly focused on two main questions: firstly, to what extent do concurrent helminth infections affect the ability of a host to control other infectious diseases and, secondly, what effect do helminth infections have on the elicitation of effective responses to vaccines?

In the case of *F. hepatica*, there is convincing data that infection with this trematode results in altered anti-bacterial immune responses in ruminants: *F. hepatica* infection in cattle confers susceptibility to *Salmonella dublin* as a consequence of parasite-induced suppression of Th1-type immunity [2], whereas peripheral blood mononuclear cells (PBMC) from cattle experimentally coinfected with *Mycobacterium bovis* and *F. hepatica* secrete reduced levels of IFN-γ in response to stimulation with mycobacterial antigens compared to PBMC from animals infected with *M. bovis* alone [29]. The latter observation is particularly significant as, in addition to increasing susceptibility to *M. bovis* infections, such modulation of the anti-*M. bovis* immune response may also reduce the capacity of currently used diagnostic tests to identify *M. bovis*-infected cattle. Currently, detection of *M. bovis* infection relies on either production of *M. bovis*-specific IFN-γ from whole blood cells or measuring delayed-type hypersensitivity reactions to *M. bovis* antigens. Experimental studies of cattle infected with *M. bovis* Bacille Calmette Guerin (BCG) demonstrate that coinfection with *F. hepatica* infections results in both reduced *M. bovis* specific IFN-γ responses and delayed-type hypersensitivity responses compared to those infected with BCG alone [26]. These observations have been confirmed in the field, where a recent epidemiological survey of 3,026 UK dairy herds has shown a significant negative association between exposure to *F. hepatica* (as determined by levels of *F. hepatica-*specific antibodies in bulk milk tank samples) and diagnosis of Bovine TB [12].

While there is clear evidence from human, rodent and pig studies that nematode infections can impact on vaccine responsiveness and susceptibility to other infectious diseases [77, 92], for ruminant species the data is less clear. Early studies of *O. ostertagi* infections in cattle identified a transient non-specific suppression of cellular immune responses during the early stages of infection [15, 85, 97, 98], suggesting that infections with this nematode result in general impairment of adaptive host immunity. Consistent with this observation, antibody responses to *Brucella abortus* and Infectious Bovine Rhinotracheitis virus (IBRV) vaccines were slower to develop in cattle coinfected with *O. ostertagi* [97], although these results were not replicated in a subsequent trial using mixed *O. ostertagi* and *Cooperia onchophora* infections [98]. More recently, it was shown that while neutralising antibody responses to a multivalent modified-live virus vaccine containing IBRV were not impaired in calves dual-infected with *O. ostertagi* and *Cooperia* spp., infected calves showed greater increases in rectal temperature following subsequent IBRV challenge compared to calves treated with anthelmintic drugs either at the time of vaccination or 2 weeks beforehand [79], suggesting that active infection with these parasites may have reduced the ability of the calves to control infection with IBRV, possibly by modulating cell-mediated immunity. In a separate study in adult dairy cows, no association was found between serum levels of *O. ostertagi*-specific antibodies and the antibody response to an inactivated rabies vaccine [11]. Data on small ruminant nematodes are lacking, although it has been shown that PBMC from sheep experimentally infected with *T. circumcincta* produce reduced levels of IFNγ and increased levels of IL-10 in response to mitogenic stimulation during later stages of infection, suggesting that infection with this nematode may result in a generalised suppression of Th1 immunity and establishment of more regulatory immune responses [52].

## Conclusions

There is a significant and growing body of evidence indicating that helminth parasites of ruminants are effective at modulating host immunity, although there is a paucity of published information on nematode and cestode parasites of ruminants compared to that for trematodes. The increasing availability of genomic sequence information for ruminant helminths (e.g. [46, 80]), advances in proteomics and the increasing availability of immunological reagents for ruminant species (http://www.umass.edu/vetimm/ and http://www.immunologicaltoolbox.co.uk/), will aid the identification of novel immunomodulatory molecules produced by these parasites, facilitating the search for novel vaccines and therapeutics. As helminth immune modulation undoubtedly involves multiple immunomodulatory molecules which target many aspects of the host immune response, it is likely that vaccines targeting multiple rather than single parasite immunomodulators will be more successful. It is also possible that combining both immunomodulatory proteins and those parasite molecules which are the targets of developing protective immune responses within the same vaccine, may result in more effective protection. The mechanism for this would be through direct immunological targeting of molecules which are responsible for critical functions within the parasite (such as feeding and reproduction) combined with the enhancement of anti-parasite responses via neutralisation of parasite immune modulation – the “double whammy”. The impact of these parasites on host immune competence as a result of bystander immune modulation is of increasing interest and concern, and considerably more work is required to understand how and to what extent these parasites affect the ability of ruminant hosts to control concurrent disease or to mount effective responses to vaccination.
